# Gene therapy using IL-27 ameliorates Sjögren's syndrome-like autoimmune exocrinopathy

**DOI:** 10.1186/ar3925

**Published:** 2012-07-24

**Authors:** Byung Ha Lee, Wendy C Carcamo, John A Chiorini, Ammon B Peck, Cuong Q Nguyen

**Affiliations:** 1 Department of Oral and Maxillofacial Diagnostic Sciences, College of Dentistry, University of Florida, Gainesville, FL 32610, USA; 2 Department of Oral Biology, College of Dentistry, University of Florida, Gainesville, FL 32610, USA; 3 Molecular Physiology and Therapeutics Branch, National Institutes of Dental and Cranial Research, National Institutes of Health, Bethesda, MD 20892, USA; 4 Center for Orphan Autoimmune Disorders, College of Dentistry, University of Florida, Gainesville, FL 32610, USA; 5 Department of Pathology, Immunology & Laboratory Medicine, College of Medicine, University of Florida, Gainesville, FL 32610, USA; 6 Department of Infectious Disease and Pathology, College of Veterinary Medicine, Gainesville, FL 32608, USA

## Abstract

**Introduction:**

Sjögren's syndrome (SjS) is a systemic autoimmune disease characterized by decreased salivary and lacrimal gland secretions, resulting in severe dry mouth and dry eyes. Recent studies have suggested that T_H_17 cells and its signature cytokine IL-17 are involved in the underlying pathogenic mechanisms leading to destructive inflammation and autoimmunity. In the present study, we examined whether IL-27, a natural inhibitor of T_H_17 activity, could down-regulate or reverse SjS in C57BL/6.NOD-*Aec1Aec2 *mice, a model of primary-SjS.

**Methods:**

Recombinant serotype 2 adeno-associated viral (AAV2) vectors expressing either *IL-27 *(rAAV2-*IL2*7) or *LacZ *(rAAV2-*LacZ*) were injected into 6 or 14 week-old C57BL/6.NOD-*Aec1Aec2 *mice. Changes in IL-27, IL-17, and IL-10 cytokine levels in peripheral blood were determined by ELISAs, while flow cytometry analyses were used to quantify cytokine-positive splenocytes. Histological assessment of salivary glands, anti-nuclear autoantibody (ANA) staining, and stimulated saliva flow rates were used to profile SjS disease severity.

**Results:**

Mice systemically treated with intravenous rAAV2-*IL27 *injections at either 6 or 14 weeks of age exhibited long-term elevated levels of serum IL-27 with concomitantly reduced levels of IL-17 compared with sera from mice injected with rAAV2-*LacZ *or saline out to 20 weeks post-inoculation. Most importantly, disease profiles revealed that rAAV2-*IL27 *treatment had little effect on lymphocytic focus (LF) scores, but resulted in structural changes in LF, lower titers of ANAs with changes in staining patterns, and a less severe clinical disease as determined by saliva flow rates.

**Conclusions:**

These data support the concept that IL-27, when provided exogenously, can induce a suppressive effect on SjS development and thus may be an effective therapeutic agent for regulating T_H_17 pro-inflammatory activity in autoimmune diseases where the T_H_17 system has been shown to play an important role in their pathogenesis.

## Introduction

Interleukin 27 (IL-27), along with IL-12, IL-23, and IL-35, is a novel cytokine of the IL-6/IL-12 family. It is composed of two subunits: IL-12p40-related Epstein-Barr virus-induced gene 3 (Ebi3) protein and IL-12p35-related p28 protein (p28) [1]. The orphan cytokine receptor WSX-1 (TCCR) and glycoprotein-130 (gp130) make up the heterodimeric signal transducing receptor for IL-27 [2]. IL-27 acts on CD4^+ ^T cells and plays a pivotal role as both a pro- and anti-inflammatory cytokine. As a pro-inflammatory cytokine, IL-27 activates T helper 1 (T_H_1) responses in the early phases of immunity, in which secretion of interferon-gamma (IFN-γ) is one of the key inflammatory mediators in autoimmunity. The mechanism appears to be the activation of signal transducer and activator of transcription 1 (STAT1) [3]. As an anti-inflammatory protein, IL-27 suppresses IL-2, antagonizing IL-6 function and activating expression of suppressor of cytokine signaling (SOCS) protein(s) [4]. In studies with WSX^-/- ^receptor knockout mice, abnormal signal transduction of IL-27 showed hyper-production of various pro-inflammatory cytokines such as tumor necrosis factor-alpha (TNF-α) and IL-6 when challenged by *Trypanosoma cruzi *or *T. gondii *[5,6]. Moreover, IL-27 can suppress the expression of forkhead box P3-positive (Foxp3^+^) regulatory T (T_reg_) cells and act as a negative regulator of human neutrophil function [7,8]. Recent studies also confirmed that IL-27 has anti-tumor effects [7,9].

IL-27 is well known for its inhibitory effects on retinoic acid-related orphan receptor gamma t (RORγt), the transcription factor for T_H_17 cells, by activating both T-bet, the transcription factor for T_H_1 cells, and the STAT1 pathway, thus inhibiting expression of IL-17A (commonly referred to as IL-17) [10]. In addition, WSX-1-deficient mice showed greater susceptibility for experimental autoimmune encephalomyelitis (EAE) in comparison with wild-type control mice and exhibited increased levels of IL-17 [11]. More recent reports have described the capacity of IL-27 to suppress T_H_17 cells by inhibiting T_H_17 cell differentiation, thereby reducing severity of T_H_17-mediated autoimmune diseases [11,12].

Gene delivery using recombinant adeno-associated virus (rAAV)-based vectors has been shown to convey long-term gene expressions in treated hosts [13-16]. Previous studies of gene therapy using AAV have also proven its safety and ability to elicit minimal inflammatory responses in comparison with other types of gene delivery agents [17-20]. Nevertheless, to date, no study using the rAAV system has reported a role for IL-27 in Sjögren's syndrome (SjS). Thus, we examined the effects of IL-27 treatment on SjS disease of C57BL/6.NOD-*Aec1Aec2 *mice when delivered either at 6 weeks of age (pre-disease) or at14 weeks of age (clinical disease). Results reported here indicate that IL-27, a potent inhibitor of T_H_17 cell development, may be a useful reagent for treating SjS.

## Materials and methods

### Animals

C57BL/6.NOD-*Aec1Aec2 *mice were bred and maintained under specific pathogen-free conditions in the animal facility of Animal Care Services at the University of Florida (Gainesville, FL, USA). Development of the C57BL/6.NOD-*Aec1Aec2 *mouse and its SjS-like disease phenotype is described elsewhere [21]. Briefly, C57BL/6.NOD-*Aec1Aec2 *mouse was developed by introducing two genetic regions, one on chromosome 1 (designated *Aec2*) and the second on chromosome 3 (designated *Aec1*), derived from the NOD/LtJ mouse, into the SjS-non-susceptible C57BL/6J mouse. All animals were maintained on a 12-hour light-dark schedule and provided food and acidified water *ad libitum*. At times indicated in the article, mice were euthanized by cervical dislocation after deep anesthetization with isoflurane, and their organs and tissues were freshly harvested for analyses. All experiments and analyses described in this article were performed with male and female mice. The breeding and the use of C57BL/6.NOD-*Aec1Aec2 *mice for gene therapy study as described here were approved by the University of Florida's Institutional Animal Care and Use Committee and Institution Biosafety Committee.

### Recombinant AAV2-IL27 (rAAV2-*IL27*) vector construction

The serotype 2 adeno-associated viral vector (AAV2) was designed to contain a cytomegalovirus (CMV)/chicken-β-actin hybrid promoter, an internal ribosome entry site (IRES), and the inverted terminal repeat sequences (pTR-UF14) (Figure S1A of Additional file 1). To fully recapitulate the functionality of mouse IL-27 cytokine, a rAAV2-*IL27 *vector was constructed by inserting the genes encoding the two subunits of IL-27 (Ebi3 and p28) into a pTR-UF14 vector. *Ebi3 *and *p28 *were generated by polymerase chain reaction (PCR) from murine cDNA by using two pairs of primers (*Ebi3*: forward 5'-AAACTAGTAGGTCCTTCCCTGGGGCCAGGT-3' and reverse 5'-TTTGATATCAAGGATCCAGTCCCTCTTCAG-3' and *p28*: forward 5'-AAAAAGCGGCCGCATGGGCCAGGTGACAGGA-3' and reverse 5'-TTTGTCGAC TTAGGAATCCCAGGCTGAGCC-3'). Ebi3 and p28 subunits flanked by IRES component were constructed allowing the co-expression of IL-27 subunits driven by the same promoter (Figure S1B of Additional file 1). We used AAV2 vector encoding beta galactosidase (rAAV2-*LacZ*) as an AAV2 control vector, whose construction was previously reported [22].

### Functional activity of IL-27 expressed from the rAAV2-*IL27 *vector

rAAV2-*IL27 *and its backbone (pTR-UF14) plasmids were transfected separately into HEK 293 cells by using TransIT-293 Transfection Reagent (Mirus Bio LLC, Madison, WI, USA) in accordance with the instructions of the manufacturer. After transfection, cell pellet and culture media were collected at different time points up to 48 hours, and expression of the two subunits of IL-27 was measured. Ebi3 was determined by Western blotting by using anti-mouse Ebi3 antibody (Santa Cruz Biotechnology, Santa Cruz, CA, USA), and p28 was detected by enzyme-linked immunosorbent assay (ELISA) by using a Quantikine kit for mouse IL-27 p28 (R&D Systems, Minneapolis, MN, USA). Biological activity was determined by flow cytometry. Culture supernatants from rAAV2-IL27 or empty AAV2-transfected HEK293 cells were collected (mock). C57BL/6J splenocytes were incubated with mock, IL-6/transforming growth factor-beta (IL-6/TGF-β) for optimal T_H_17 differentiation, and IL-6/TGF-β with supernatants of AAV2-IL27 transfected cells at 37°C for 5 days. IL-17^+ ^cells were measured by flow cytometry.

### Packaging of recombinant AAV2 (rAAV2) vector particles

To generate rAAV2 viral particles, adenoviral helper packaging plasmid pDG was used. Plates (15 cm) with less than 40% confluent HEK 293T cells were co-transfected with either pAAV2-*LacZ *(control vector) or rAAV2-*IL27 *in accordance with standardized methods [23]. Clarified cell lysates were adjusted to a refractive index of 1.372 by the addition of CsCl and centrifuged at 38,000 revolutions per minute (rpm) for 65 hours at 20°C. Equilibrium density gradients were fractionated, and fractions with a refractive index of 1.369 to 1.375 were collected. The particle titer was quantified by real-time PCR, and the vector was stored at -80°C.

### Injections of vectors

Two different age groups of C57BL/6.NOD-*Aec1Aec2 *mice were used to take advantage of the pre-disease and clinical disease stages of the animal [24] for the IL-27 gene therapy, thereby aiming to either prevent the onset of the disease or reverse the disease phenotype of SjS (6-week-old mice for pre-disease stage and 14-week-old mice for clinical disease stage, respectively). Mice were treated systemically with saline or rAAV2-LacZ or rAAV2-IL27, and there were 10 mice (five male and five female) per treatment for each disease stage. Systemic delivery was done via intravenous injection in tail veins. Mice were anesthetized with a ketamine (100 mg/mL, 1 mL/kg body weight; Fort Dodge Animal Health, Fort Dodge, IA, USA) and xylazine (20 mg/mL, 0.7 mL/kg body weight; Phoenix Scientific, St. Joseph, MO, USA) cocktail by intraperitoneal injection. Then 50 μL of vector solution containing 2 × 10^10 ^vector genome (VG) was injected. Control groups received either the same amount of rAAV2-LacZ VG or the same volume of saline. The dosage was chosen on the basis of published studies by others for extensive study of dosage optimization [25-29]. All animals were sacrificed at 20 weeks after delivery. At the time of euthanization, systemic transgene expression was determined by using quantitative real-time PCR (Figure S2 of Additional file 2).

### Intracellular cytokine staining and flow cytometry analysis

Single-cell suspensions of spleen cells were prepared from C57BL/6.NOD-*Aec1Aec2 *mice as described elsewhere [30] and at the ages designated in the text. In brief, spleens were freshly explanted, gently minced through stainless steel sieves, suspended in phosphate-buffered saline (PBS), and centrifuged (1,200 rpm for 5 minutes). Erythrocytes were lysed by a 7-minute incubation in 0.84% NH_4_Cl. The resulting leukocyte suspensions were washed two times in PBS, counted, and re-suspended in culture media (RPMI 1640 medium, 10% fetal bovine serum, 2 mM L-glutamine, and 0.05 mM β-mercaptoethanol) to a density of 2 × 10^6 ^cells/mL. One million cells were pipetted to individual wells of a 24-well microtiter plate pre-coated with anti-CD3 (10 μg/mL; BD Pharmingen, San Diego, CA, USA) and anti-CD28 (2 μg/mL; BD Pharmingen) antibodies for T-cell activation. Cells were incubated for 5 hours with Leukocyte Activation Cocktail containing GolgiPlug (2 μL/mL; BD Pharmingen). Collected cells were fixed and permeabilized by using a Cytofix/CytopermFixation/Permeabilization kit (BD Pharmingen). The flow cytometry acquisition for intra- and extra-cellular staining was performed following staining with fluorescein isothiocyanate (FITC)-conjugated anti-mouse IL-27 (R&D Systems), phycoerythrin (PE)-conjugated anti-IL-17 (eBioscience, San Diego, CA, USA), and PE-Cy7-conjugated anti-mouse CD4 (Invitrogen, Carlsbad, CA, USA) for the detection of cytokines from gene-delivered mice. The cells were counted by using a BD ACCURI C6 Flow Cytometer (BD Biosciences, San Jose, CA, USA) and analyzed by FlowJo software (TreeStar Inc., Ashland, OR, USA). CD3^+ ^and CD4^+ ^T cells were gated, and the cells were analyzed for IL-27^+ ^or IL-17^+ ^cells.

### Cytokine level of peripheral blood

Measurement of IL-27levels in sera samples was conducted by using a Quantikine kit for mouse IL-27 p28 (R&D Systems). IL-17, and IL-10 levels are detected using mouse IL-17 and IL-10 ELISA kits (Life Technologies, Grand Island, NY, USA). All procedures were performed in accordance with the instructions of the manufacturers. Readings were carried out by using a Model 680 Microplate Reader (Bio-Rad, Hercules, CA, USA).

### Hematoxylin and eosin staining and histological assessment

Salivary glands, lacrimal glands, liver, lung, and kidney were surgically removed from each C57BL/6.NOD-Aec1Aec2 mouse at the time of euthanasia and placed in 10% phosphate-buffered formalin for 24 hours. Fixed tissues were embedded in paraffin and sectioned at a thickness of 5 μm. Paraffin-embedded sections were deparaffinized by immersing in xylene, followed by dehydration in ethanol. The prepared tissue sections were stained with hematoxylin and eosin (H&E) dye (Histology Tech Services, Gainesville, FL, USA). H&E-stained sections were observed under a microscope for glandular structure and leukocyte infiltration. Lymphocytic foci (LF) were enumerated by three individuals in blinded fashion. LF were defined as aggregates of more than 50 leukocytes quantified per each histological section, and adjacent sections were used for immunofluorescent staining. Stained sections were observed at ×6 magnification by using a Leica MZ8 microscope, and images were obtained with Leica application suite (version 2.4.0.R1; Leica, Wetzlar, Germany).

### Immunofluorescence staining

Tissue sections were deparaffinized, rehydrated, and blocked with hydrogen peroxide. For antigen retrieval, tissue sections were heated to 100°C under pressure with 10 mM citrate buffer for 15 minutes. The sections were incubated with rat anti-mouse B220 (BD Pharmingen, San Diego, CA, USA) or goat anti-mouse CD3 (Santa Cruz Biotechnology, Inc, Santa Cruz, CA, USA) to detect B cells and T cells, respectively, overnight at 4°C. The sections were washed two times with PBS-Tween and incubated with Texas Red-conjugated rabbit anti-rat IgG (Biomeda, Foster City, CA, USA) for B220 marker and FITC-conjugated rabbit anti-goat IgG (Sigma-Aldrich, St. Louis, MO, USA) for CD3 detection for 30 minutes at room temperature. After additional washes, the slides were treated with mounting medium with 4'-6-diamidino-2-phenylindole (DAPI) (Vector Laboratories, Burlingame, CA, USA). Stained sections were observed at ×100 magnification with a Zeiss Axiovert 200 M microscope, and images were obtained with AxioVs40 software (version 4.7.1.0; Carl Zeiss, Jena, Germany).

### Detection of anti-nuclear antibodies in the sera

Anti-nuclear antibodies (ANAs) in the sera of mice were detected by using an HEp-2 ANA kit (Inova Diagnostics, Inc., San Diego, CA, USA). All procedures were performed in accordance with the instructions of the manufacturer. In brief, HEp-2-fixed substrate slides were overlaid with appropriate mouse sera diluted 1:50, 1:100, 1:200, 1:400, and 1:800. Slides were incubated for 1 hour at room temperature in a humidified chamber. After three washes for 5 minutes with PBS, the substrate slides were covered with Alexa 488-conjugated goat anti-mouse IgG (H/L) (Invitrogen) diluted 1:100 for 45 minutes at room temperature. After three washes, fluorescence was detected by fluorescence microscopy at ×200 magnification by using a Zeiss Axiovert 200 M microscope, and all images were obtained with AxioVs40 software (version 4.7.1.0; Carl Zeiss) with a constant exposure of 0.3 seconds (Carl Zeiss). In this study, three individuals in blinded fashion examined positive staining patterns.

### Measurement of stimulated saliva flow

To measure stimulated saliva flow, each mouse was weighed and injected with a cocktail of isoproterenol (0.2 mg/mL) and pilocarpine (0.5 mg/mL) in 100 μL of saline by intraperitoneal injection. Saliva was collected for 10 minutes from the oral cavity of individual mice by using a micropipette starting 1 minute after injection of the secretagogue. The volume of each saliva sample was measured. Baseline saliva flow rates (SFRs) were measured a week before the gene therapy, and SFRs were measured every 4 weeks after 8 weeks of post-gene delivery periods until the end of the experiment in each group.

### Statistical analyses

The data are expressed as the mean ± standard error of the mean. Statistical evaluation was determined by using one-way analysis of variance test with the GraphPad Prism software (GraphPad Software, Inc., La Jolla, CA, USA) or chi-squared test. A *P *value of less than 0.05 considered statistically significant.

## Results

### rAAV2-*IL27 *plasmid is highly efficient in producing a functional IL-27 product *in vitro*

**Figure 1:**
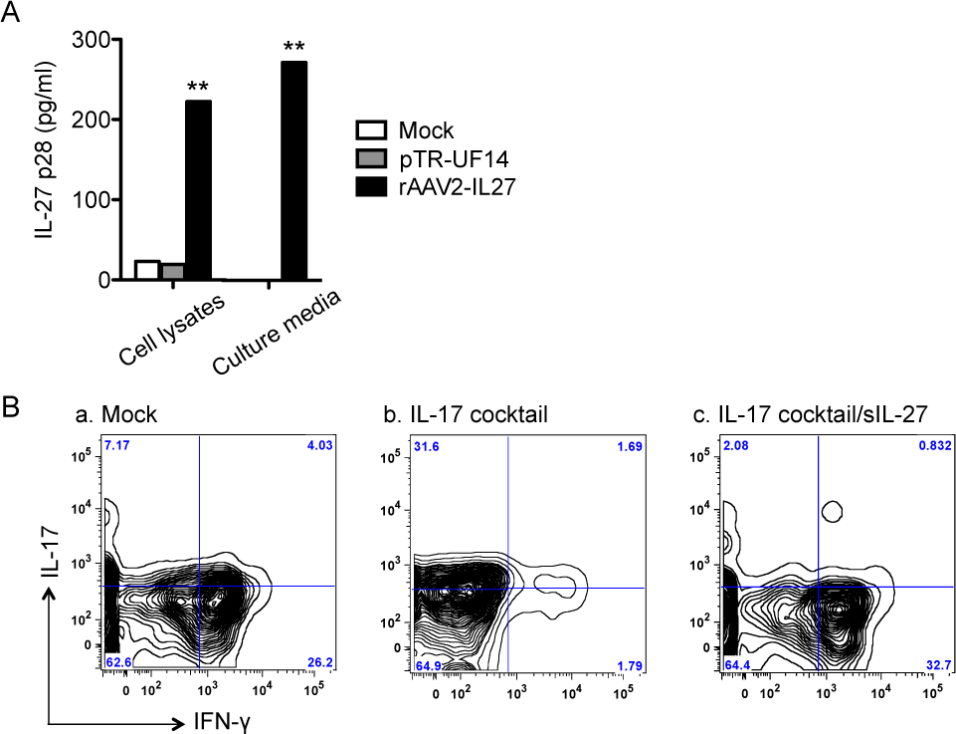
**Generation of functional IL-27-expressing vector (rAAV2-*IL27*)**. **(A) **IL-27 p28 expression levels in the cell lysates or culture media of mock transfection which contain transfection reagents only (white bar) or control empty AAV2 plasmid (grey bar) or rAAV2-IL27 (black bar) transfected HEK293 cells at 48 hours after transfection. **(B) **Biological effects of IL-27 were generated from rAAV2-IL27 on C57BL/6 splenocytes by using media only or **(a) **mock, **(b) **IL-17 differentiation cocktail containing IL-6 and transforming growth factor-beta, or **(c) **IL-17 differentiation cocktail with supernatant IL-27 from rAAV2-IL27 transfected HEK293 cells (sIL-27). The experiment was done in triplicate and repeated three times for consistency. Values in bar graphs are mean. **P < 0.01, rAAV2-*IL27 *group versus mock or rAAV2 transfected by one-way analysis-of-variance test. IFN-γ, interferon-gamma; IL, interleukin.

To determine functional activity of the rAAV2-*IL27 *plasmid construct, HEK293 cells were transfected with either the rAAV2-*IL27 *or its backbone, pTR-UF14 plasmid. To control for background, untreated HEK293 cells were cultured in medium containing the transfection reagents only (representing a mock transfection). Forty-eight hours after transfection, cell lysates were prepared and culture media were collected to quantify expression levels by ELISA of both secreted and non-secreted IL-27 protein. As presented in Figure 1A, IL-27 p28 levels were significantly increased in lysates from cells transfected with rAAV2-*IL27 *(222 pg/mL) in comparison with lysates from either the mock transfected (24 pg/mL) or pTR-UF14 transfected (22 pg/mL) cells. Similarly, examination of culture media showed elevated levels of IL-27 p28 (272 pg/mL), but IL-27 p28 was not detected in either culture media of mock transfected or pTR-UF14 transfected HEK293 cells. Analysis of the cell lysates and culture media also indicated that Ebi3 expression was highly increased in rAAV2-*IL27 *transfected cells (data not shown).

Biological activity of recombinant IL-27 (rIL-27) secreted by rAAV2-*IL27 *was determined by using culture media supernatants collected from the rAAV2-*IL27 *and control by supernatants from pTR-UF14 transfected HEK293 cells or cells alone. C57BL/6J splenocytes cultured in media containing IL-6 and TGF-β supplemented with supernatants containing rIL-27 showed significant decreases in IL-17^+ ^cells with concomitant increases in IFN-γ^+ ^cells in comparison with media supplemented with supernatant from either pTR-UF14 or mock transfected cells (Figure 1B). Considered together, these results indicate that the heterodimeric rIL-27 protein is properly expressed by the rAAV2-*IL27 *vector and is biologically functional in suppressing T_H_17 cells *in vitro*.

### Decreased serum IL-17 levels in mice following rAAV2-*IL27 *treatment

**Figure 2:**
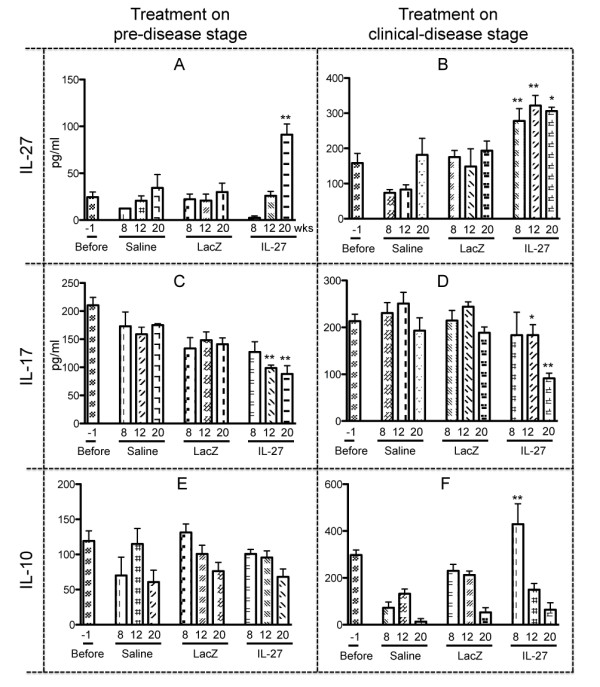
**Changes in circulating cytokines in sera following rAAV2-*IL27 *treatment**. Ten mice (five male and five female) were treated systemically with saline (negative control) or rAAV2-*LacZ *(vector control) or rAAV2-*IL27 *per treatment for each disease stage (6-week-old mice for pre-disease stage and 14-week-old mice for clinical disease stage). Each mouse was injected with 2 × 10^10 ^vector genome of rAAV2-*IL27 *or rAAV2-*LacZ *in 50 μL of saline via intravenous line in tail veins. Sera were collected 1 week before (-1, baseline) and 8, 12, and 20 weeks after the treatment of pre-disease and clinical disease mice groups. Levels of cytokines (IL-27, IL-17, and IL-10) were detected by enzyme-linked immunosorbent assay. IL-27 levels in pre-disease (**A**) and clinical-disease (**B**) stages. IL-17 levels in pre-disease (**C**) and clinical-disease (**D**) stages. IL-10 levels in pre-disease (**E**) and clinical-disease (**F**) stages. Values are mean ± standard error of the mean (*n *= 7). *P < 0.05 rAAV2-*IL27 *group versus rAAV2-*LacZ *or saline groups by one-way analysis-of-variance test; **P < 0.01. IL, interleukin; IL-27, rAAV2-*IL27*-treated groups; LacZ, rAAV2-*LacZ*-treated groups; Saline, saline-treated groups.

To determine the systemic cytokine levels of IL-27 in C57BL/6.NOD-*Aec1Aec2 *mice following treatment with rAAV2-*IL27*, rAAV2-*LacZ *(vector control), or saline (negative control), sera of treated mice were examined 8, 12, and 20 weeks after gene delivery. As indicated in Figure 2A, 6-week-old mice inoculated with rAAV2-*IL27 *exhibited significant increases in IL-27 levels during the subsequent 20-week observation period, resulting in a threefold increase in comparison with the rAAV2-*LacZ*- or saline-treated control groups (91 versus 34 and 30 pg/mL, respectively). High levels of circulating IL-27 were concomitantly correlated with IL-17 level decreases observed at both 12 and 20 weeks after injection (Figure 2C). Interestingly, IL-10 levels were unaffected by the increased levels of IL-27 (Figure 2E).

Similar, yet more pronounced, results were observed in mice administered rAAV2-*IL27 *at 14 weeks of age, the time of adaptive immunity onset in the C57BL/6.NOD-*Aec1Aec2 *mice. At 8 weeks after injection, mice injected with rAAV2-*IL27 *showed dramatic increases in the levels of IL-27 in comparison with the rAAV2-*LacZ*- or saline-treated control groups (278 versus 175 and 173 pg/mL, respectively), and these levels were maintained for at least 20 weeks after injection (Figure 2B). Concomitantly, rAAV2-*IL27*-treated mice showed reduced levels of IL-17 at both 12 and 20 weeks after vector delivery in comparison with the rAAV2-*LacZ*- and saline-treated control groups (Figure 2D), indicating that decreased expression of IL-17 in the sera is correlated with the increased expression of IL-27 in the rAAV2-*IL27*-treated mice. In contrast to mice treated at 6 weeks of age, IL-10 was highly elevated at 8 weeks after injection in mice treated with rAAV2-*IL27 *in the clinical disease phase (Figure 2F). These data suggest a direct negative regulation of T_H_17 cells with a corresponding upregulation of IL-10 production, which could further inhibit cytokine productions from T_H_1, T_H_2, and T_H_17 cells. Thus, the two disease stages of SjS respond differently to IL-27 treatment.

### Intravenous injection of rAAV2-*IL27 *negatively regulates splenic T_H_17 cell functions

**Figure 3:**
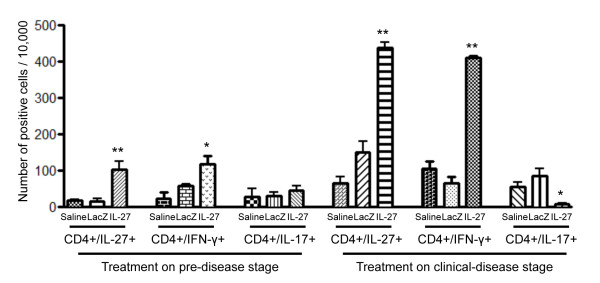
**rAAV2-IL27-expressing vector is capable of negatively regulating splenic T_H_17 cells**. Splenocytes were isolated from each mouse and stained for CD3 and CD4 fluorochrome-conjugated antibodies. Cells were further intracellularly stained for IL-27, IL-17, and IFN-γ. Flow cytometry analysis was performed with cells gated on CD4^+ ^populations. Values are mean ± standard error of the mean (*n *= 7). *P < 0.05 rAAV2-*IL27 *group versus rAAV2-*LacZ *or saline groups by one-way analysis-of-variance test; **P < 0.01. IFN-γ, interferon-gamma; IL, interleukin; IL-27, rAAV2-*IL27*-treated groups; LacZ, rAAV2-*LacZ*-treated groups; Saline, saline-treated groups.

As mentioned previously, IL-27 functions to inhibit the activity of T_H_17 cells by negatively regulating the production of IL-17 while directly promoting the production of IFN-γ by T_H_1 cells. Since rAAV2-*IL27 *was administered intravenously, splenocytes were isolated to examine the systemic effects of the vector on T_H_17 and T_H_1 cells. Splenocytes from mice treated at either 6 or 14 weeks of age were isolated 20 weeks after treatment to profile the levels of IL-27, IFN-γ, and IL-17 produced by specific T-cell populations. As presented in Figure 3, rAAV2-*IL27*-injected mice showed relative increases in the number of CD3^+^CD4^+^IL-27^+ ^cells, determined by flow cytometry analyses, in comparison with mice treated with rAAV2-*LacZ *vector or saline. The increases in IL-27^+ ^T cells were associated with increases in IFN-γ^+ ^cells. Although rAAV2-*IL27 *treatment of mice at 6 weeks of age resulted in increased numbers of IL-27^+ ^T cells, there were no differences in IL-17^+ ^T cells between saline-, rAAV2-*LacZ*-, and rAAV2-*IL27*-treated groups, and this was possibly due to the overall low numbers of IL-27^+ ^T cells. In contrast, mice injected with rAAV2-*IL27 *at 14 weeks of age had decreased numbers of IL-17^+ ^T cells with a concomitant two- to fourfold increase in IL-27^+ ^T cells over rAAV2-*LacZ*- and saline-treated groups (Figure 3). These data are consistent with the concept that systemic delivery of IL-27 can regulate the levels of IL-17^+ ^T cells in the spleen, but high levels of exogenous IL-27 may needed to suppress the levels of IL-17.

### Lymphocytic infiltrates in the exocrine glands of rAAV2-*IL*27-injected mice

Lymphocytic infiltration present in the salivary glands is one of the main diagnostic markers for SjS. In the C57BL/6.NOD-*Aec1Aec2 *mouse model, the development and onset of autoimmunity in the exocrine glands are considered the results of acinar cell apoptosis that signals an influx of leukocytes expressing pro-inflammatory cytokines. Salivary gland lymphocytic infiltrates initially are composed of T-cell clusters followed by recruitment of B lymphocytes [31,32]. The migration of specific T-cell populations secreting IL-17 and IL-23 has been shown to directly contribute to the pathogenesis of the disease [33]. Therefore, to determine the effect of rAAV2-*IL27 *treatment on lymphocytic infiltration in the salivary glands of mice injected at 6 or 14 weeks of age with rAAV2-*IL27*, freshly explanted, formalin-fixed, paraffin-embedded salivary gland sections were examined after euthanizaton at 20 weeks after treatment.

**Figure 4:**
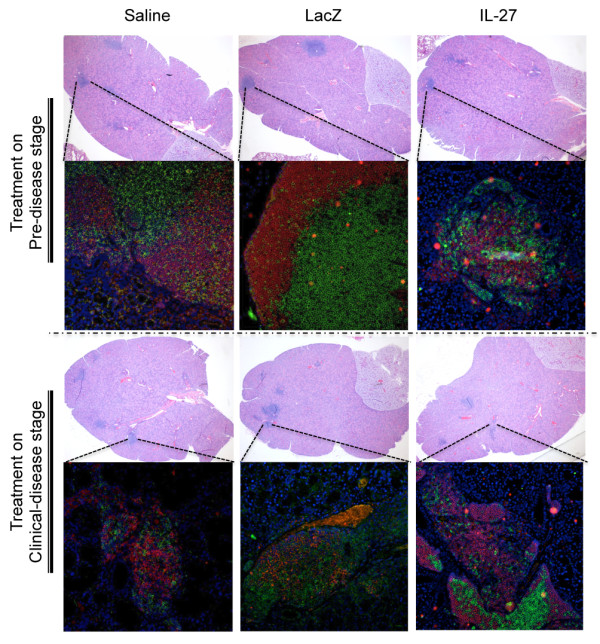
**Lymphocytic infiltrates in the salivary glands**. Representative images for hematoxylin and eosin (H&E) and CD3/B220 staining for saline-, rAAV2-LacZ-, and rAAV2-IL27-treated groups at 20 weeks after gene delivery in pre-disease and clinical disease groups. H&E images were taken at ×6 magnification and immunofluorescence staining images of lymphocytic infiltrates with anti-CD3 to detect T cells (green) and anti-B220 to detect B cells (red) were taken at ×100 magnification with a Zeiss Axiovert 200 M microscope. IL-27, rAAV2-*IL27*-treated groups; LacZ, rAAV2-*LacZ*-treated groups; Saline, saline-treated groups.

Representative images of salivary gland infiltrates, shown in Figure 4, reveal the presence of LF composed of both B and T lymphocytes, whereas the numbers of LF in the salivary glands were scored for each experimental group (Table 1). As can be seen, rAAV2-*IL27*-treated mice showed no significant reductions in the number of LF in the salivary glands in comparison with either rAAV2-*LacZ*- or saline-treated mice, irrespectively of the age at which treatment of the mice was carried out. In contrast, lacrimal glands of the rAAV2-*IL27*-treated mice were noted to have a decrease in the numbers of LF in comparison with saline-treated mice, whereas lacrimal glands explanted from mice treated with rAAV2-*LacZ *showed slightly lower LF scores. Furthermore, in general, larger infiltrates were found in the rAAV2-*LacZ*- and saline-treated groups with higher numbers of B cells in comparison with rAAV2-*IL27*-treated mice. Owing to the intravenous injection of the viral vectors, other organs such as liver, lungs, and kidney were also examined for lymphocytic infiltration. However, no significant pathological changes were observed between mice belonging to the different treatment groups (data not shown). These results indicate that systemic injection of rAAV2-*IL27 *has a positive impact on LF scores in the lacrimal glands, especially when treatment is initiated at 6 weeks of age, but appears to have little or no impact on LF scores in salivary glands.

**Table 1:** Quantification of lymphocytic infiltrates in the salivary glands of gene-delivered mouse groups

Disease stage		Salivary glands		Lacrimal glands	
	Treatment	LF	Mean^a^	LF	Mean
	Saline	5/6^b ^(83%)^c^	2.3 ± 1.5	4/6 (67%)	2.8 ± 1.3

Pre-disease	Lac-Z	7/7 (100%)	3.0 ± 2.4	6/7 (86%)	1.5 ± 0.7
	IL-27	6/7 (86%)	4.3 ± 2.2	2/7^d ^(29%)	2.0 ± 1.4

Clinical disease	Lac-Z	7/7 (100%)	3.4 ± 2.4	4/7 (57%)	2.5 ± 1.0
	IL-27	7/7 (100%)	3.4 ± 1.3	2/7 (29%)	4.5 ± 5.0

^a^Mean lymphocytic foci (LF) score of each gland in mouse ± standard error of the mean per histological salivary gland section; ^b^number of mice; ^c^percentage of mice; ^d^*P *< 0.05 by chi-squared test. IL-27, rAAV2-*IL27*-treated groups; LacZ, rAAV2-*LacZ*-treated groups; saline, saline-treated groups.

### Intravenous injection of rAAV2-*IL-27 *treatment alters the anti-nuclear antibody profile in C57BL/6.NOD-*Aec1Aec2 *mice

**Figure 5:**
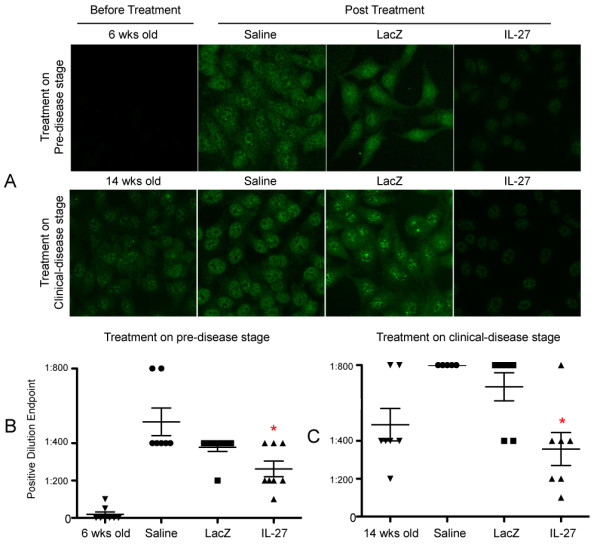
**Nuclear speckled anti-nuclear antibody staining pattern profile**. **(A) **Representative images of nuclear speckled anti-nuclear antibody at a dilution of 1:400. Sera were collected in the pre-disease group at 6 weeks of age (before treatment) and 26 weeks of age or 20 weeks after treatment. In the clinical disease group, sera were collected at 14 weeks of age (before treatment) and 34 weeks of age or 20 weeks after treatment. Images were captured at ×200 magnification with a Zeiss Axiovert 200 M microscope with a constant exposure of 0.3 seconds. **(B) **Nuclear speckled titers in pre-disease treated mice. **(C) **Nuclear speckled titers in clinical disease treated mice. Three individuals in blinded fashion determined autoantibody titers. Values are mean ± standard error of the mean (*n *= 7). *P < 0.05 rAAV2-*IL27 *group versus rAAV2-*LacZ *or saline groups by one-way analysis-of-variance test. IL-27, rAAV2-*IL27*-treated groups; LacZ, rAAV2-*LacZ*-treated groups; Saline, saline-treated groups. These symbols have no specific meanings because the legends on the x-axis defined them. However, if we must define these symbols, are you ok with? Pre-disease stage: 6 weeks old mice (inverted triangle), saline-treated mice (circle), rAAV2-*LacZ*-treated mice (square), rAAV2-*IL27*-treated mice (triangle). Clinical-disease stage: 14 weeks old mice (inverted triangle), saline-treated mice (circle), rAAV2-*LacZ*-treated mice (square), rAAV2-*IL27*-treated mice (triangle).

Patients with SjS often develop ANAs, specifically anti-Ro (SS-A) or anti-La (SS-B) or both. ANAs that generally show a specked staining pattern when HEp-2 cells are used are highly prevalent in SjS, and the titers of such ANAs are clinically important factors in diagnosing the disease. To identify the presence of ANAs, sera collected from C57BL/6.NOD-*Aec1Aec2 *mice treated at either 6 or 14 weeks of age with rAAV2-*IL27*, rAAV2-*LacZ*, or saline were tested for ANA reactivity by using HEp-2 cells. Antibody titers were examined at dilutions of 1:50, 1:100, 1:200, 1:400, and 1:800. As presented in Figure 5A, sera from mice injected with rAAV2-*IL27 *at either age showed comparatively weak or no nuclear speckled antibody staining on the HEp-2 cells (shown at a dilution of 1:400), whereas mice injected with AAV2-*LacZ *or saline showed strong ANA staining, and this was predominantly a nuclear speckled pattern. Quantification of the nuclear speckled ANA titers clearly revealed that, in mice treated at either age, the titers present in rAAV2-*IL27*-treated mice were significantly lower than in mice receiving injections of rAAV2-*LacZ *vector or saline (Figure 5B, C). Thus, these results suggest that increased levels of IL-27 in sera following rAAV2-*IL27 *treatment directly affect production or profiles of autoantibodies (or both) in SjS mice.

### Measurement of stimulated saliva flow rates

**Figure 6:**
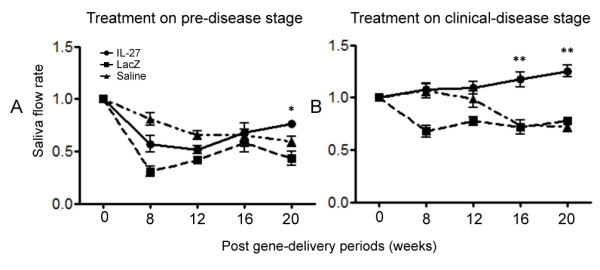
**Measurement of stimulated saliva flow rates (SFRs)**. One week prior to treatment, saliva was collected and measured to determine the baseline SFRs. SFRs for each time point (8, 12, 16, and 20 post-gene delivery periods) were normalized against the baseline SFRs indicated as zero (0) post-gene delivery periods. **(A) **Gene delivery in pre-disease stage at 6 weeks of age. **(B) **Gene delivery in clinical disease stage at 14 weeks of age. Values are mean ± standard error of the mean (*n *= 7). *P < 0.05 rAAV2-*IL27 *group versus rAAV2-*LacZ *or saline groups by one-way analysis-of-variance test; **P < 0.01. IL-27, rAAV2-*IL27*-treated groups; LacZ, rAAV2-*LacZ*-treated groups; Saline, saline-treated groups.

To compare SFRs in the C57BL/6.NOD-*Aec1Aec2 *mice injected with rAAV2-*IL27*, rAAV2-*LacZ*, or saline, pilocarpine/isopreterenol-stimulated saliva collections were carried out every 4 weeks starting 8 weeks after treatment, and each value equated to the average baseline SFRs collected from the mice 1 week prior to beginning treatment. As presented in Figure 6, mice treated with either rAAV2-*IL27 *or rAAV2-*LacZ *at 6 weeks of age exhibited a relatively rapid loss in SFRs. Mice in the rAAV2-*IL27 *treatment group, however, showed a slight recovery of SFRs with time, whereas mice in the rAAV2-*LacZ *treatment group remained low. As expected, mice receiving saline showed a progressive loss in SFRs. These results suggest that the viral vectors *per se *can influence the SFRs and that IL-27 may be able to reverse the effect, albeit weakly.

In contrast to the above results, mice treated with either rAAV2-*IL27 *or rAAV2-*LacZ *at 14 weeks of age exhibited different responses to the treatments (Figure 6B): mice receiving the rAAV-*LacZ *vector again exhibited a rapid loss of SFR, whereas mice receiving the rAAV2-*IL27 *vector slowly showed minor improvements in their SFRs. Mice receiving saline began showing a slower SFR after about 8 weeks after treatment (or 22 weeks of age), consistent with disease in this model. These results indicate that IL-27 might impact the delayed recovery of SFRs when treatment is done prior to the onset of disease and may partially restore saliva secretion and glandular function at a later stage.

## Discussion

This study was undertaken to examine the effects of IL-27 gene therapy in SjS by using the C57BL/6.NOD-*Aec1Aec2 *mouse model of primary SjS. SjS is characterized by hyposalivation, autoantibodies in sera, and lymphocytic infiltration in the salivary glands. Previous findings also suggested a role for the IL-23/T_H_17/IL-17A system in both patients with SjS and animal models [33,34]. Therefore, we hypothesized that IL-27 would have a therapeutic role in treating SjS and show potential as a therapeutic for the human disease. The results of the present study generally support our hypothesis, showing that IL-27 overexpression by the rAAV2-*IL27 *vector in C57BL/6.NOD-*Aec1Aec2 *mice increased saliva secretion and decreased ANA formation despite a lack of differences in LF scores between rAAV-*IL27*- and rAAV2-*LacZ *vector-treated mice or saline-treated controls injected at 6 or 14 weeks of age. Interestingly, rAAV2-*IL27 *treatment at the early clinical disease stage appeared to show a better response than treatment at a pre-disease stage.

The role of IL-27 has been studied, but the underlying mechanism of IL-27 in autoimmune disease, specifically SjS, is still unknown. Although IL-27 has pro-inflammatory functions, which are embodied in activation of IFN-γ production from T_H_1 cells, many recent studies are showing anti-inflammatory effects of IL-27 against microorganisms and effector T cells, including T_H_17 cells. Recent studies have also shown that IL-27 is involved in anti-inflammatory functions, especially interfering with TNF-α or T_H_17 cells (or both) in autoimmune diseases, including multiple sclerosis, rheumatoid arthritis, and systemic lupus erythematosus [35-38]. For the potential therapeutic use of IL-27, previous *in vivo *and *in vitro *studies have shown that IL-27 can be used as an anti-tumor or anti-viral agent as well as a modulator of autoimmunity [39-43]. IL-27-expressing vectors have been shown to exhibit potent anti-tumor activity in malignancies and metastatic tumors [9,44,45]. Therefore, the present study, using IL-27 in a gene therapy protocol, provides evidence that supports its potential effectiveness in autoimmunity.

Our previous study, in which an adenoviral vector was used, demonstrated that overexpression of IL-17 was capable of recapitulating SjS phenotypes (lowered saliva secretion and increased autoantibodies) in normal C57BL/6J mice and, at the same time, showed differential immunological or biological responses depending on the age of mice at the time of treatment [34]. By inducing long-term expression of IL-27 levels by using AAV2, our data showed marked reductions in IL-17 cytokine levels in blood from rAAV2-*IL27*-injected mice at two different phases of disease onset. Interestingly, there was delayed expression of IL-27 in rAAV2-IL27-treated mice in the innate immune phase in comparison with the expression in the adaptive immune phase. This phenomenon could be attributed to the age of the animals at the time of vector treatment. A study by Bostick and colleagues [46] showed that AAV9 transduction by systemic delivery could be influenced by the age of the animals. These authors found that AAVs exhibited different transduction efficiencies and that adult mice showed higher transduction efficiency than newborn mice. Furthermore, other studies have implicated the 'time lag' effect of AAV2-mediated transgene expression in various tissues, specifically myocardium [47-49]. Zincarelli and colleagues [50] categorized AAV2 as a low-expression and slow-onset virus along with AAV3, 4, and 5 among AAV serotypes 1 to 9 after systemic injection in mice. Our study also showed that the regulation of IL-17 could be directly attributed to the overexpression of an IL-27 transgene, but this could be the indirect systemic effect of IL-27 on other regulatory cytokines and cell types. Although serum IL-27 is elevated at 8, 12, and 20 weeks after treatment in the 14-week treated group, serum IL-17 is markedly decreased at 20 weeks after treatment. A number of factors can regulate the function of IL-17, especially T_H_1 cells releasing IFN-γ [4], which was increased in the rAAV2-*IL27*-treated group (Figure 3). Also, independent roles of IL-27 subunits, p28 and Ebi3, might be critical. A recent study by Stumhofer and colleagues [51] showed that IL-27 p28 alone binds to gp130 as a pro-inflammatory signal antagonist. Although gp130 is the subunit for IL-27 receptor with WSX-1 (IL-27Rα), it is also a subunit for IL-6 receptor, which is involved in T_H_17 cell differentiation. In addition, Ebi3, a cytokine subunit of IL-35, is known for its suppression of T_H_17 cells [52]. Although rAAV2-*IL27 *vector expresses both p28 and Ebi3, the possibility of independent roles of p28 or Ebi3 could not be excluded here. Lastly, other cell types (for instance, Tr-1) can regulate T_H_17 cells. IL-27 has been shown to be able to stimulate Tr-1 cells, which release IL-10 through STAT1 and STAT3 signaling [53,54]. IL-10 is a cytokine that is known for its anti-inflammatory function and the inhibitory effect on T_H_17 cells. However, recent studies using IL-27-overexpressing transgenic mice showed an antagonist role of IL-27 on T_reg _cells, another source for IL-10, through IL-2 modulation [55]. Thus, IL-27 may have an effect on the balance of Tr-1 cells and T_reg _cells in the mouse or human system. Therefore, multi-factorial regulation of IL-17 might have contributed to the data in this study.

One unique feature of SjS is the formation of leukocyte aggregates in the salivary and lacrimal glands, referred to as either lymphoepithelial sialadenitis or LF [56,57]. At times, these LF appear histologically to be germinal-like centers and may be indicative of a more pronounced and severe hypergammaglobulinemia [58]. Although LF and LF scores are important criteria for clinical disease and depict the level of lymphocytic infiltrations of the exocrine glands, the scores often do not correlate with the severity of disease [59,60]. Until recently, it was thought that salivary gland-infiltrating leukocytes were mostly B lymphocytes and CD4^+ ^T_H_1 cells. However, we recently reported that LF contain significant numbers of CD4^+ ^T_H_17 memory T cells plus IL-23-producing macrophages or dendritic cells or both [33]. Although the present study shows no difference in mean LF scores between IL-27-treated and AAV2-*LacZ *vector- or saline-treated controls at the innate or adaptive immune phase, other research has suggested a poor correlation between this marker of disease and salivary gland function [61].

As might be anticipated, only treatment with rAAV2-*IL27 *at the clinical disease stage showed a stable and gradual increase of SFRs compared with baseline SFRs over the course of the experiment, whereas mice treated with rAAV2-*IL27 *at the pre-disease stage showed slight recovery of glandular function at 20 weeks after treatment. These data are consistent with the serum IL-17 levels, which were significantly downregulated at 20 weeks after treatment for both studied groups. Further investigations are needed to determine the underlying mechanism of how IL-27 influences SFRs, although one possible mechanism is the direct suppression of IL-17 in promoting the formation of spontaneous germinal centers [62], thereby dampening pathogenic autoantibody production [63]. This concept appears to be supported in part by the altered ANA profile and significant decrease in autoantibody titers in rAAV2-*IL27*-treated mice.

## Conclusions

We have provided a proof of concept that exogeneous IL-27 is a potential factor for treating SjS. Although additional research in regard to gene delivery methods and optimal disease time points are needed to further validate the use of rAAV gene transfer, the use of mouse models and systemic delivery of IL-27 provides a unique system to study treatment of autoimmunity at the clinical disease stage, which is applicable for treating patients with established disease. Our results suggest that IL-27 gene therapy could be an effective therapeutic strategy to reverse the symptoms of SjS and should be further investigated.

## Abbreviations

AAV2: serotype 2 adeno-associated viral vector; AEC: autoimmune exocrinopathy; ANA: anti-nuclear autoantibody; EAE: experimental autoimmune excephalomyelitis; Ebi3: Epstein-Barr virus-induced gene 3; ELISA: enzyme-linked immunosorbent assay; FITC: fluorescein isothiocyanate; gp130: glycoprotein-130; H&E: hematoxylin and eosin; IFN-γ: interferon-gamma; IL: interleukin; IRES: internal ribosome entry site; LF: lymphocytic foci; PBS: phosphate-buffered saline; PCR: polymerase chain reaction; PE: phycoerythrin; rAAV: recombinant adeno-associated virus; rpm: revolutions per minute; SFR: saliva flow rate; SjS: Sjögren's syndrome; STAT: signal transducer and activator of transcription; TGF-β: transforming growth factor-beta; T_H_: T helper; TNF-α: tumor necrosis factor-alpha; T_reg_: regulatory T; VG: vector genome.

## Competing interests

The authors declare that they have no competing interests.

## Authors' contributions

BHL performed vector construction, gene therapy, saliva collections, and disease profiling of the mice and participated in the design of the study, data analyses, and manuscript preparation. WCC carried out histological analysis and ANA staining. JAC helped with AAV2 packaging, study design, and manuscript preparation. ABP and CQN participated in the design of the study, data analyses, and manuscript preparation. All authors read and approved the final manuscript.

## Supplementary Material

Additional file 1 **Figure S1. Generation of IL-27 expressing serotype 2 adeno-associated viral vector (AAV2-IL27)**. A: Diagram of pTR-UF14 vector, which was given by Dr. Sergi Zolotuhkin (Department of Pediatrics, University of Florida College of Medicine) for the back-bone structure of rAAV2-IL27. B: Diagram of rAAV2-IL27. To fully recapitulate the functionality of mouse IL-27 cytokine, a rAAV2-IL27 vector was constructed by inserting the genes encoding the two subunits of IL-27 (Ebi3 and p28) into a pTR-UF14 vector. Click here for file

Additional file 2 **Figure S2. Relative expression of IL-27 p28 mRNA in liver**. To compare the transgene expression, livers in each group were collected at the end of experiments (20-week of post delivery periods) and total RNAs were extracted for cDNA synthesis. PCR primers were designed using IDT's PrimerQuestSM (Integrated DNA Technologies Inc., Coralville, IA, USA). Quantitative realtime PCR was performed using the iCycler IQTM multi-color realtime PCR detection system (Bio-Rad Laboratories). The Ct values obtained were normalized to those of 18S ribosomal RNA. Level of IL-27 p28 mRNA in LacZ or IL-27 delivered group were normalized against the mRNA level in saline group. (values are mean ± SEM, *p < 0.05 rAAV2-IL27 group versus rAAV2-LacZ or saline groups by one-way ANOVA test). Click here for file
