# Supplementary figures and images for: Gene therapy using IL-27 ameliorates Sjögren's syndrome-like autoimmune exocrinopathy
Source: Arthritis Res Ther. 2012 Jul 24;14(4):R172. doi: 10.1186/ar3925 (PMC3580566; doi:10.1186/ar3925)

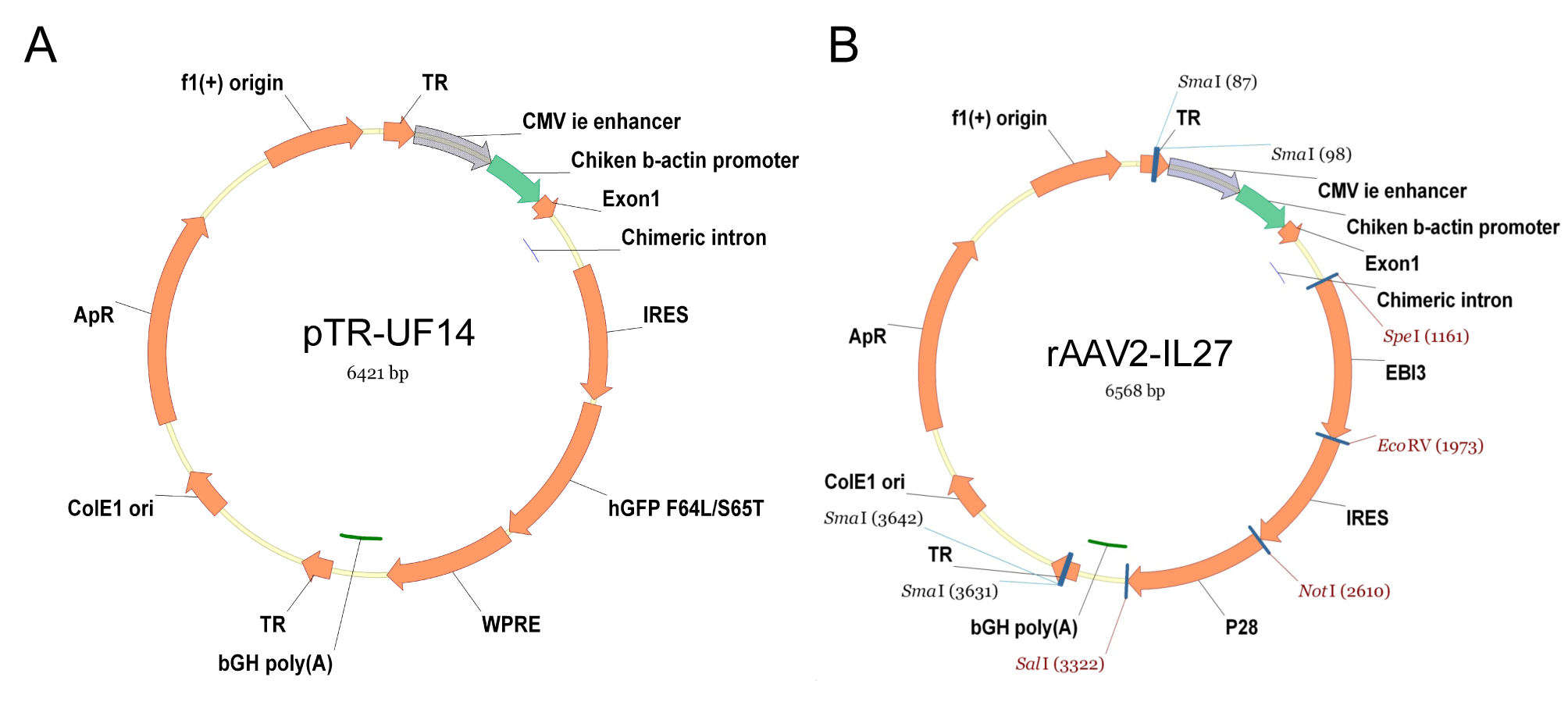

Supplement: Additional file 1 — Figure S1. Generation of IL-27 expressing serotype 2 adeno-associated viral vector (AAV2-IL27). A: Diagram of pTR-UF14 vector, which was given by Dr. Sergi Zolotuhkin (Department of Pediatrics, University of Florida College of Medicine) for the back-bone structure of rAAV2-IL27. B: Diagram of rAAV2-IL27. To fully recapitulate the functionality of mouse IL-27 cytokine, a rAAV2-IL27 vector was constructed by inserting the genes encoding the two subunits of IL-27 (Ebi3 and p28) into a pTR-UF14 vector. [file ar3925-S1.TIFF]

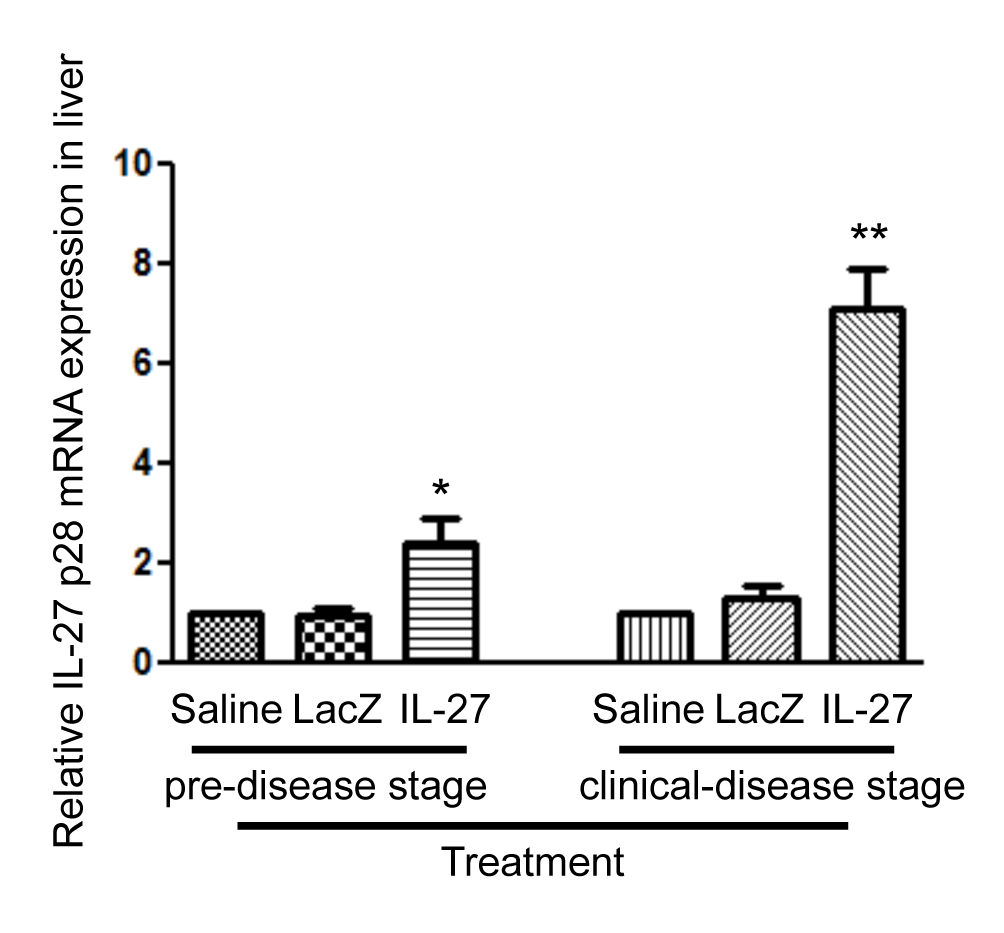

Supplement: Additional file 2 — Figure S2. Relative expression of IL-27 p28 mRNA in liver. To compare the transgene expression, livers in each group were collected at the end of experiments (20-week of post delivery periods) and total RNAs were extracted for cDNA synthesis. PCR primers were designed using IDT's PrimerQuestSM (Integrated DNA Technologies Inc., Coralville, IA, USA). Quantitative realtime PCR was performed using the iCycler IQTM multi-color realtime PCR detection system (Bio-Rad Laboratories). The Ct values obtained were normalized to those of 18S ribosomal RNA. Level of IL-27 p28 mRNA in LacZ or IL-27 delivered group were normalized against the mRNA level in saline group. (values are mean ± SEM, *p < 0.05 rAAV2-IL27 group versus rAAV2-LacZ or saline groups by one-way ANOVA test). [file ar3925-S2.TIFF]
